# Impact of Thyroid Hormone Levels on Functional Outcome in Neurological and Neurosurgical Early Rehabilitation Patients

**DOI:** 10.1155/2017/4719279

**Published:** 2017-08-16

**Authors:** Melanie Boltzmann, Simone B. Schmidt, Jens D. Rollnik

**Affiliations:** Institute for Neurorehabilitation Research, BDH-Clinic Hessisch Oldendorf, Hannover Medical School, Hannover, Germany

## Abstract

**Background:**

Neurological and neurosurgical early rehabilitation (NNER) is a specialized treatment option for patients with severe neurological disorders. The present study investigated whether thyroid hormone levels on admission have an impact on the outcome of NNER patients.

**Method:**

The study included 500 NNER patients who were admitted to the BDH-Clinic Hessisch Oldendorf between 2009 and 2010. Data such as age, sex, diagnoses, comorbidities, Glasgow Coma Scale score, length of stay, and thyroid hormone levels (obtained as part of clinical routine care) were analyzed retrospectively. Improvement in the Early Rehabilitation Barthel Index (ERBI) at the end of the NNER treatment was defined as outcome parameter.

**Results:**

Most patients made functional progress during treatment, as reflected in significant enhancements of the ERBI. Approximately half of the patients were transferred to further rehabilitation treatment. Young age, early onset of NNER treatment, low functional impairment on admission, and, in particular, low total T3 levels were independently associated with a good outcome.

**Conclusion:**

Age, severity of disease, and time between injury and admission are known to predict outcome. The present study confirms the influence of these general factors. In addition, an association between thyroid hormones and functional outcome was demonstrated for NNER patients.

## 1. Introduction

Acute critical events induce complex multisystem reactions, which also affect endocrine functions [1]. From the endocrine spectrum, alterations of thyroid-related hormone concentrations have been reported for different neurological disorders, that is, ischemic stroke (e.g., [2–4]), traumatic brain injury (TBI, e.g., [5, 6]), and intracerebral hemorrhage (e.g., [7–9]). There is limited evidence that serum levels of thyroid-related hormones might influence functional outcome in the acute phase of brain damage. However, the relationship is complex, and the relevance for functional outcome and the question of therapeutic interventions remain the subject of an ongoing debate [1].

In stroke, the most common abnormality in thyroidal hormone levels is a reduction in serum triiodothyronine (T3), which is associated with disease severity and functional outcome [2–4, 10, 11]. In a study investigating ICU patients, a low free T3 level was found to be the strongest predictor of mortality among the thyroid hormones [12]. For patients with acute traumatic brain injuries, thyroid hormone deficiencies (e.g., low levels of T3, T4, and TSH) are reported in 2 to 15 percent of cases [5]. While reductions of free T3 and TSH are associated with higher mortality and poor short-term prognosis, free T4 is not reliably related to outcome. Results from Zetterling and colleagues indicate that the association between low T3 levels and poor short-term functional outcome in stroke and TBI patients can also be found after intracerebral hemorrhages [7]. Low T3 levels are present in at least one-third of patients with acute intracerebral hemorrhage [9], while TSH deficiencies were described in less than two percent of patients [8].

In short, a low T3 level is a common finding after critical illnesses and seems to be associated with poor outcomes [13, 14]. The low T3 concentration refers to total T3 as well as free T3 [7]. Most likely, the low T3 concentration in the acute phase after the critical event is caused either by decreased peripheral transformation of T4 to T3 or by increased turnover of thyroid hormones. It is assumed that cytokines, endogenous thyroid hormones, low concentrations of binding proteins, and increased amounts of free fatty acids and bilirubin inhibit hormone binding and metabolism [15, 16]. Some of the drugs used in acute care or rehabilitation facilities such as glucocorticoids, iodinated contrast agents, propranolol, barbiturates, dopamine, opiates, furosemide, and sulfonamides disrupt the thyroid gland at different levels and may trigger an iatrogenic inhibition of thyroid function [7]. Data regarding the relationship between free T4 or TSH and outcome are not as clear. The main reason might be that only few studies have examined TSH, T3, and T4 values concurrently [3]. However, there is some evidence indicating that deficiencies in T4 and TSH are related to functional outcome as well [3, 5, 10]. The long-term dynamics of endocrine alterations after severe brain injury has not been sufficiently investigated yet. Thus, the present study examines the prognostic value of different thyroid hormones concurrently (T3, T4, and TSH) in a sample of postacute patients undergoing neurological and neurosurgical early rehabilitation (NNER).

## 2. Methods

### 2.1. Data Collection

The study was conducted at the BDH-Clinic Hessisch Oldendorf, a large specialized neurological rehabilitation facility in Germany. The clinic offers treatment in all phases of neurological rehabilitation, ranging from acute patient care (phase A) to medical-occupational rehabilitation (phase E). Furthermore, the clinic has a special focus on neurological and neurosurgical early rehabilitation (phase B). The phase model of neurological rehabilitation in Germany is explained in [17].

For the study, routine data from 558 patients were retrospectively reviewed. The patients were admitted to the clinic between January 2009 and December 2010. Baseline characteristics including demographic data, diagnoses, comorbidities, Glasgow Coma Scale (GCS) score, length of stay, thyroid hormone levels, and Activity of Daily Living (ADL) assessment were recorded for each patient. For the ADL assessment, the Early Rehabilitation Barthel Index was used [18], which is composed of the Barthel Index (BI) and the Early Rehabilitation Index (ERI). The BI is one of the most common measures in neurological rehabilitation. The functional independence of the patients is assessed with ten ordinal-scaled items resulting in a scale of 0 to 100 (with 0 being completely dependent and 100 being completely independent). The ERI, on the other hand, measures the presence of the following criteria: intensive care supervision, tracheostoma, mechanical ventilation, orientation disorder, behavioral disorder with endangerment of self and others, severe impairment of communication, and swallowing disorder. If a criterion is fulfilled, −25 points (heavy communication disorder) or −50 points (all other criteria) are assigned (range: −325 to 0 points). The sum of the BI and the ERI results in the ERBI, with a range from −325 to 100. A prerequisite for inclusion in phase B is an ERBI value ≤ 30. For monitoring the functional outcome, ERBI was documented on admission, after four weeks of inpatient treatment and at discharge. In order to control the influence of the length of stay, gains in ADL measures (value at discharge minus value on admission) were divided by the length of stay. Patients with known altered thyroidal functions (e.g., hyperthyroidism, hypothyroidism, Hashimoto's diseases, and thyroidectomy) and/or using medication affecting thyroid function like thyroxine, levothyroxine, thiamazole, or iodide (*n* = 58) were excluded from the study.

Blood samples were collected from patients within 24 hours after admission to the rehabilitation facility. Serum levels of thyroid stimulating hormone (TSH), total triiodothyronine (tT3), and free thyroxine (fT4) were measured. Normal ranges of hormone levels are 0.7 to 1.9 ng/ml for tT3, 0.6 to 1.5 ng/dl for fT4, and 0.34 to 4.82 mU/l for TSH. On the basis of the thyroid profile, patients were divided into three categories: patients with serum levels within the normal ranges are considered to be “euthyroid.” Any value of tT3, fT4, or TSH below 0.7, 0.6, and above 4.82, respectively, are taken as “hypothyroid.” Values of tT3, fT4, or TSH above 1.5, 1.9, and below 0.34, respectively, are considered as “hyperthyroid.”

### 2.2. Patients

500 patients undergoing NNER treatment were enrolled in the study (216 female, 284 male). The average age of all patients was 65 years (SD = 15 years, Md = 69 years, and range: 16 to 92 years). The most common diagnosis was stroke (38.6 percent), followed by intracerebral hemorrhage (21.8 percent) and traumatic brain injury (18.2 percent). Other diagnoses, such as hypoxic brain damage, neoplasms, inflammatory diseases, spinal traumata, and diseases of the peripheral nervous system, were summarized in the category “other” due to their low number of cases (21.4 percent). Prior to NNER treatment, the patients were treated for an average of 22 days in an acute care hospital (SD = 18 days, Md = 18 days, and range: 1 to 202 days). The NNER treatment lasted on average 43 days (SD = 28 days, Md = 39 days, and range: 3 to 194 days). Approximately 91 percent of the patients were treated continuously, while the other 9 percent had at least one interruption with a mean duration of 8 days (SD = 5 days, Md = 7 days, and range: 1 to 24 days). The main reasons for treatment interruptions were transfers to acute care hospitals, especially for implantations of shunts or drug pumps, replacement of bone flap, or treatment of complications. The amount of time that passed from the acute event until the admission to the rehabilitation facility was on average 23 days (SD = 24 days, Md = 19 days, and range: 0 to 286 days). 112 patients (22.4 percent) were supplied with a tracheostoma and 37 patients (7.4 percent) were mechanically ventilated on admission. In particular, these two criteria are indicators of disease severity in phase B patients.

### 2.3. Statistical Analyses

For statistical analyses, the SPSS 23.0 software package was used. Since most of the data was not normally distributed, nonparametric statistical methods were used.

In univariate analyses, group differences were evaluated with the* Mann–Whitney U test* (comparison of two groups). Differences between outcome measures on admission, after four weeks, and at discharge were tested with the nonparametric* Wilcoxon test* for dependent samples. Partial correlation controlling for age and gender was used to examine linear relationships. Multivariate analyses were used to examine which factors predict the functional outcome. Specifically, a multiple linear regression model was used to predict gains in ERBI values at discharge.

Categorical data are presented as proportions, and group differences were assessed with *χ*^2^-tests. For non-normally distributed data, the median (Md) is specified in addition to the mean (M) and standard deviation (SD). For graphical representations, mean and standard errors (SE) are used. Differences were regarded as significant with *p* < .05.

## 3. Results

The proportions of patients considered as either euthyroid, hypothyroid, or hyperthyroid according to their levels of total T3, free T4, and TSH are shown in Table 1. The TSH level was negatively correlated with the free T4 level (*r*_*s*_ = −0.109; *p* < .05), but not with the total T3 level.

On admission, no associations between thyroid hormones and functional measures were found. At discharge, however, patients with higher total T3 levels exhibited significantly higher gains in the ERBI than patients with lower total T3 levels (*r*_*s*_ = 0.187; *p* < .001). There was no association between functional outcome and free T4 level or TSH level.

In a next step, subanalyses for individual main diagnoses were performed. For stroke patients, low free T4 levels on admission were associated with higher gains in functional outcome (*r*_*s*_ = −0.169; *p* = .019). TBI patients were more likely to have good functional outcome when the total T3 concentration was high on admission (*r*_*s*_ = 0.342; *p* < .01). In patients with intracerebral hemorrhages, thyroid hormone concentrations were not related to functional outcome.

**Figure 1:**
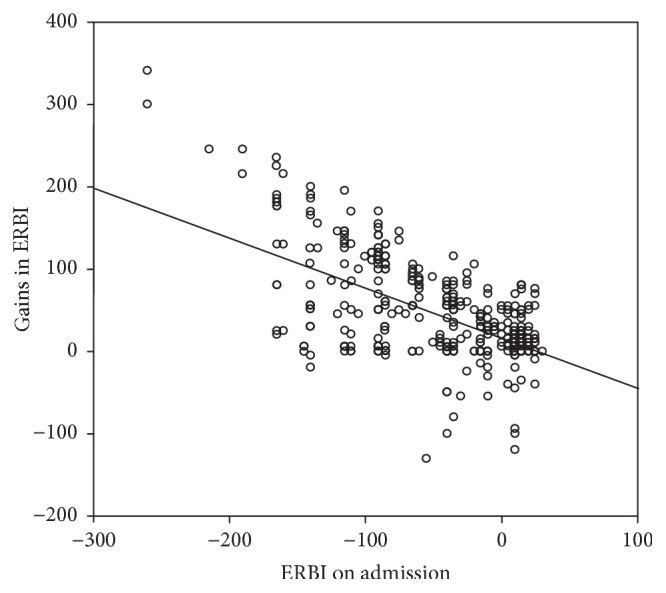
Relationship between ERBI score on admission and gains in ERBI score during treatment.

Patients had an initial ERBI value of −38.73 (SD = 58, Md = −23, and range: −260 to 30). After four weeks of inpatient treatment, the value increased by 16 points (*Z* = −10.08; *p* < .001) and by further 23 points until the time of discharge (*Z* = −9.88; *p* < .001). As Figure 1 illustrates, the lower the ERBI was on admission, the greater the improvement was during NNER treatment (*r*_*s*_ = −0.254; *p* < .001).

Using models of stepwise multiple linear regression, effects of thyroid hormone levels and other variables known to be associated with gains in functional outcome (ERBI) were investigated. In the multiple model (*R*_adj._^2^ = 0.330; *p* < .001), the ERBI value on admission had the strongest predictive value for gains in functional outcome (*β* = −0.599; *p* < .001). Other significant predictors were the GCS score and the total T3 level on admission as well as age and the time between injury and admission. However, levels of free T4 and TSH could not predict the functional outcome. The respective regression coefficients of included and excluded variables are shown in Table 2.

Out of the 500 patients, 244 (49 percent) were transferred to subsequent rehabilitation phases after NNER treatment, due to their persistent functional progress (phase C: 95.1 percent; phase D: 4.9 percent). These patients have been assigned to the “favorable functional status” group. The remaining patients did not make any further functional progress at the end of NNER, which is why the rehabilitation process was eventually terminated. In most cases, patients were transferred either to an acute care hospital (17.6 percent), to a professional care facility (55.5 percent), or to home care (17.2 percent). A further 9.8 percent of the patients died during treatment. These patients were classified in the “unfavorable functional status” group. Demographic characteristics of both groups are shown in Table 3. Patients with favorable functional status at discharge were characterized by younger age, higher GCS score, higher ERBI score, and higher total T3 levels on admission. The treatment of patients with favorable outcome was shorter and less frequently interrupted by transfers to acute care facilities. Moreover, the gains in functional outcome, as measured with ERBI, were considerably higher for patients with favorable outcome compared to patients with unfavorable outcome.

## 4. Discussion

The aim of the present study was to investigate the impact of thyroid hormone concentrations on the functional outcome in NNER. As a main result, initial (functional) impairment, age, time between injury and admission, and total T3 serum level proved to be significant predictors for functional gains, as measured with the Early Rehabilitation Barthel Index (ERBI).

Out of all investigated thyroid hormones, only total T3 was associated with functional outcome in the whole group of NNER patients. Since the effect was confirmed in a multivariate analysis controlling for effects of other known predictors, total T3 level may be considered as independent predictor of functional outcome.

Low T3 concentration and its association with poor functional outcome are well documented for critically ill patients [2, 4, 7, 10–12, 19]. In a recent study, Wang et al. reported that of all thyroid hormones T3 is the best predictor for intermediate care unit mortality [12]. Additionally, the extent of the T3 decline has been found to reflect the severity of the initial disease [2–4, 10, 11, 20]. The reduction of T3 indicates a stronger suppression of the hypothalamus-thyroid (HPT) axis in critical injuries and poor functional outcome. It is currently unclear whether this is a protective adaptation of the organism to the injury or a maladaptive response that leads to hypothyroidism on the tissue plane. It is likely that the central suppression of the HPT axis is part of the neuroendocrine adaptation to a critical disease, aiming to save energy [11, 15].

While most studies investigated free T3 concentrations, there are some studies reporting associations between low total T3 levels and poor prognosis [2, 11, 20]. Likewise, low total T3 levels being in the normal range are also associated with higher morbidity and poor functional outcome [10]. However, most studies failed to show that free or total T3 concentration has independent predictive value for functional outcome. Associations were shown only in univariate analyses (e.g., [3, 10, 21]). Only few studies confirmed that low T3 concentration has independent predictive value for poor functional outcome [4, 19]. However, since most studies investigated either the total or the free fraction of T3, further studies are necessary to determine the specific role of each fraction as predictor for functional outcome.

Besides total T3 concentration, the present study also analyzed free T4 and TSH concentration but found no influence on functional outcome in patients undergoing NNER treatment. However, subanalyses for different main diagnoses revealed an association between high free T4 concentration on admission and poor functional outcome in stroke patients. The T4 concentration usually decreases after critical injuries ([1, 13, 22]; see also [23]), but Xu and colleagues found increased T4 levels in stroke patients. Importantly, the T4 concentration was higher in patients with high NIHSS scores and poor outcome [10]. This finding might be explained by the fact that the peripheral conversion of T4 to T3 has been reduced as a result of the ischemic insult. Thus, the T3 concentration decreased, whereas the T4 concentration increased. The decrease in peripheral conversion of T3 to T4 has also been associated with the severity of the disease [10].

Several studies have shown that the concentrations of T3 are also reduced after severe traumatic brain injuries [5, 24] and intracerebral hemorrhages [7, 11]. A low concentration of (free) T3 is associated with a poor functional outcome in TBI [5] and intracerebral hemorrhages [7].

The clinical implications of the acute alterations in hormone concentrations after severe brain injuries are largely unknown. Most of the changes are likely to be adaptive physiological processes in response to the critical illness. However, the question whether hormone alterations should be substituted is the subject of a controversial debate [24]. Some studies report a neuroprotective role of preexisting clinical hypothyroidism [25, 26], leading to an extensive discussion about the question of correcting low T3 values after stroke [24].

In addition to thyroid functions, the functional recovery depends to a large extent on the severity of the disease and on the severity of the initial functional impairment [27–30]. In international studies, the Functional Independence Measure (FIM) is used as an indicator for the severity of the initial functional impairment [30, 31]. In the present study, ERBI on admission was used as equivalent measure to the FIM. With higher ERBI values on admission, patients had higher ERBI values at discharge and were more likely to be transferred to further inpatient rehabilitation. Patients with low initial ERBI values were prone to a higher risk of poor outcome. This might be due to the fact that patients who continued to meet certain criteria of the ERI (e.g., swallowing disorder, tracheostomy, and mechanical ventilation) would have to improve significantly in the BI in order to compensate for the deficits of the ERI. Only under those conditions, gains in functional outcome would result in higher ERBI values. With respect to changes of the ERBI, a negative correlation with the initial ERBI was found. Patients with low initial values made more progress during rehabilitation treatment than patients with high initial values. This relationship can be explained by the scaling of the ERI, which is included in the ERBI. In the case that a patient meets a certain criterion of the ERI, a negative value of 50 points is added to the sum score. As soon as the criterion will no longer apply, 50 points are withdrawn. The rough scaling of the ERI and the fact that most patients did not meet most ERI criteria at the end of NNER (e.g., 53 percent withdrawal of tracheal cannula rate, 76 percent weaning rate) might explain the significant gains in the ERI, and thus the ERBI.

Overall, the findings of the present study are in line with other studies, showing an association between severe initial impairment and poor prognosis [30, 32, 33].

Another significant predictor for functional outcome was the age of the patients. Younger patients were more likely to have a good functional outcome. For stroke, several studies confirm that younger age is associated with a better outcome (see review [34]). Age effects are reported in the NNER literature for both, the BI [33] and the ERI [35]. Additionally, patients made more functional progress when the NNER treatment was initiated early after the injury. A similar association is reported for stroke patients. Here, the greatest therapeutic success is achieved within the first 30 days after stroke onset [27], but an early onset within 24 hours may also adversely affect the outcome [36]. However, this relationship is influenced by the severity of the disease. The admission of severely impaired patients to NNER treatment is often delayed, due to medical complications [30].

## 5. Limitations

There are some limitations to our study, which should be considered. First, data have been collected retrospectively; thus only available routine data could be analyzed. In the future, data should be collected prospectively, ensuring that all relevant influencing factors are recorded. Secondly, we used the ERBI as functional outcome measure. Although the ERBI has a better change sensitivity than the BI in assessing severely impaired neurological patients [32], it also has some shortcomings. The focus of the ERBI is on ADL assessment and on criteria relevant for critically ill NNER patients (e.g., the presence of a tracheostoma and/or mechanical ventilation). In future studies, cognitive abilities should be taken into account as well, as is the case for the FIM [37]. Thirdly, thyroid hormone levels had been measured only once at the beginning of the NNER treatment. In future studies, blood samples should be collected repeatedly in order to monitor changes in thyroid hormone concentrations over time. Finally, levels of TSH, total T4, free T4, total T3, and free T3 should be measured concurrently. Due to these limitations, the results of the present study should be considered cautiously.

## 6. Conclusion

The present study aimed to identify predictors for functional outcome of patients undergoing NNER. Of special interest was the question, whether thyroid hormone levels on admission have a predictive value for prognosis. Therefore, total T3, free T4, and TSH serum levels were measured concurrently. In a mixed sample of neurological and neurosurgical diseases (e.g., stroke, traumatic brain injury, and intracerebral hemorrhage), age, severity of initial (functional) impairment, time between injury and admission, and total T3 level were found to be of independent predictive value for the functional outcome. Although the present study highlights the importance of thyroidal functions in the postacute phase, further studies should examine the temporal dynamics more closely.

## Figures and Tables

**Table 1:** Classification of patients according to their thyroid hormone concentrations of total T3, free T4, and TSH.

Hormone	Hypothyroid		Euthyroid		Hyperthyroid	
	*n*	%	*n*	%	*n*	%
Total T3	130	26.0	340	68.0	30	6.0
Free T4	14	2.8	454	90.8	32	6.4
TSH	51	10.2	447	89.4	2	0.4

**Table 2:** Included and excluded variables used in a linear regression model to predict gains in the Early Rehabilitation Barthel Index (ERBI).

	*Gains in ERBI*	
	*β-coefficient*	*Significance*
*Included variables*		
ERBI on admission	−0.530	0.000
GCS on admission	0.412	0.000
Total T3	0.202	0.000
Age	−0.179	0.002
Time between injury and admission	−0.176	0.002
*Excluded variables*		
Free T4	−0.013	0.980
TSH	0.005	0.984
Duration of acute treatment	−0.046	0.687

**Table 3:** Characteristics of patients with favorable and unfavorable functional status at the end of NNER treatment.

	Functional status		Significance
	Favorable (*n* = 244)	Unfavorable (*n* = 256)	
*On admission*			
** **Age (years)	61.7 (±15.2)	69.1 (±14.9)	0.000
** **ERBI	−29 (±57.8)	−48 (±56.7)	0.000
** **GCS	12.3 (±3.0)	10.2 (±3.6)	0.000
** **Time between injury and admission	22.5 (±18.2)	24.4 (±28.6)	0.663
** **Duration of acute treatment	21.9 (±13.4)	22.2 (±20.3)	0.108
** **Total T3	0.97 ± 0.461	0.95 ± 0.54	0.024
** **Free T4	1.03 ± 0.28	1.08 ± 0.37	0.110
** **TSH	1.26 ± 0.83	1.23 ± 0.94	0.305
*At discharge*			
** **ERBI	29.9 (±26.1)	−26.3 (±52.2)	0.000
** **ERBI gain	58.9 (±62.0)	21.6 (±44.2)	0.000
** **Length of stay	34.9 (±23.8)	51.7 (±29.8)	0.000
** **Number of interruptions	7.3 (±3.8)	7.8 (±5.4)	0.002

## Abbreviations

ADL: Activities of Daily Living
BI: Barthel Index
ERBI: Early Rehabilitation Barthel Index
FIM: Functional Independence Measure
HPT: Hypothalamus-thyroid axis
GCS: Glasgow Coma Scale
NNER: Neurological and neurosurgical early rehabilitation
TBI: Traumatic brain injury.
